# Regional Disparities, Economic Development, and Neonatal Mortality and Hospital Delivery in China

**DOI:** 10.1001/jamanetworkopen.2024.43423

**Published:** 2024-11-06

**Authors:** Hai Fang, Haijun Zhang, Arturo Vargas Bustamante, Shusheng Luo, Xi Chen, Yanqiu Gao, Jianmeng Liu

**Affiliations:** 1 China Center for Health Development Studies, Peking University, Beijing, China; 2 Institute of Reproductive and Child Health/National Health Commission Key Laboratory of Reproductive Health, Peking University School of Public Health, Beijing, China; 3 Department of International Health, Bloomberg School of Public Health, Johns Hopkins University, Baltimore, Maryland; 4 Department of Health Policy and Management, Fielding School of Public Health, University of California Los Angeles, Los Angeles; 5 Department of Maternal and Child Health, School of Public Health, Peking University, Beijing, China; 6 Office for National Maternal and Child Health Statistics of China, School of Public Health, Peking University, Beijing, China; 7 Department of Health Policy and Management, Yale School of Public Health, New Haven, Connecticut; 8 Department of Economics, Yale University, New Haven, Connecticut

## Abstract

**Importance:**

A negative association between neonatal mortality and hospital delivery has been found in some low- and lower-middle-income countries but not in rural settings characterized by poor quality of maternal and child health care.

**Objective:**

To examine the association between neonatal mortality and hospital delivery in China across urban and rural regions, regional disparities, and varying levels of economic development.

**Design, Setting, and Participants:**

This retrospective cohort study used county-level data from 2008 to 2020 from the National Maternal & Child Health Statistics across mainland China. Statistical analysis was conducted from March to December 2023.

**Exposures:**

Since 2008, China has strategically leveraged hospital deliveries with national subsidies to diminish neonatal mortality, particularly in rural areas.

**Main Outcomes and Measures:**

Neonatal mortality and hospital delivery rates were calculated, and their association was estimated using multivariable fixed-effects linear models of county-level cohort data to adjust for time-invariant differences across counties and controls for gross domestic product (GDP) per capita, women’s years of education, hospital beds, and health workers.

**Results:**

The analysis included data from 2930 counties, with 198.7 million live births across 36 255 county-year records between 2008 and 2020. The mean (SD) neonatal mortality rate per 1000 live births decreased in rural areas from 12.3 (7.5) in 2008 to 3.9 (2.7) in 2020 and decreased in urban areas from 5.0 (3.1) in 2008 to 2.0 (1.3) in 2020. Hospital delivery rates increased in rural areas from a mean (SD) of 93.4% (11.8%) in 2008 to 99.9% (0.6%) in 2020 and increased in urban areas from 97.7% (6.1%) in 2008 to 100.0% (0.1%) in 2020. In rural areas, an increase of 10 percentage points in hospital deliveries was associated with a neonatal mortality rate of −1.4 (95% CI, −1.9 to −1.0; *P* < .001) per 1000 live births, whereas this negative association was not observed in urban areas. When the analysis was stratified by regions and incomes, the negative association became considerably stronger in the western and central regions of China, as well as in counties with lower GDP per capita.

**Conclusions and Relevance:**

This cohort study of more than 2900 counties in China suggests that an increase in hospital deliveries was associated with reduced neonatal mortality in rural and economically underdeveloped areas in China. To further reduce neonatal mortality and improve newborn health, it is imperative to increase the accessibility of hospital delivery services.

## Key Points

**Question:**

Was neonatal mortality associated with hospital delivery between 2008 and 2020 in China across urban and rural areas, regional disparities, and varying levels of economic development?

**Findings:**

This cohort study used data from 2930 counties, with 198.7 million live births across 36 255 county-year records. A significant negative association was found between hospital delivery and neonatal mortality in rural areas of China’s western and central regions, as well as in counties with a lower gross domestic product per capita.

**Meaning:**

This study suggests that hospital delivery remains a critical public health intervention to lower neonatal mortality and enhance newborn health in China.

## Teaser

This cohort study examines the association between neonatal mortality and hospital delivery in China across urban and rural regions, regional disparities, and varying levels of economic development.

## Introduction

Globally, the number of neonatal deaths decreased from 5.0 million in 1990 to 2.3 million in 2022. This accounted for 47% of all deaths among children younger than 5 years in 2022, reflecting an increase from 40% in 1990.^1^ Worldwide, the neonatal mortality rate decreased from 36.8 per 1000 live births in 1990 to 17.3 per 1000 live births in 2022.^1^ In comparison, China experienced a more significant decrease in the neonatal mortality rate, from 29.8 per 1000 live births in 1990 to 3.0 per 1000 live births in 2022, highlighting the country’s substantial progress in improving neonatal health outcomes.^2^ The United Nations Sustainable Development Goal (SDG) 3.2.2 aims to reduce neonatal mortality to at least as low as 12 per 1000 live births by 2030.^3^ In some low- and lower-middle-income countries characterized by relatively low rates of hospital delivery coupled with high neonatal mortality rates, a negative association between hospital delivery and neonatal health outcomes had been observed in urban areas and economically developed regions.^4,5,6,7,8^ On the other hand, some studies have identified no significant association between hospital delivery and neonatal mortality in settings characterized by poor quality of maternal and child health care, particularly in rural areas and economically underdeveloped regions.^9,10,11,12,13,14^

China met the Millennium Development Goal 4 by achieving a two-thirds reduction in mortality of children younger than 5 years in 2008.^15^ Infant mortality also significantly decreased from approximately 200 per 1000 live births in 1949 to 5.6 per 1000 live births in 2019.^16^ Surveillance data have indicated that the leading causes of neonatal deaths in China are preterm birth complications and intrapartum-related events.^17^ Some of these neonatal deaths could potentially be averted with improved access to deliveries in hospitals offering comprehensive neonatal care services.

Before 2008, rural areas in China had lower hospital delivery rates and higher neonatal mortality compared with their urban counterparts.^18^ In response, a nationwide subsidy program for hospital deliveries was launched in 2008, with a particular focus on rural regions. This policy functioned as a conditional cash transfer program, with the central government providing a subsidy of ¥400 (US $1.00 = ¥6.83 in 2009) for rural residents in the western region and ¥300 for those in the central region to cover expenses related to hospital delivery. In addition, the New Cooperative Medicine Scheme, a social health insurance program in rural areas, offered supplementary reimbursements of approximately ¥300 or more for hospital deliveries. These governmental subsidies and New Cooperative Medicine Scheme reimbursements for cesarean deliveries are higher than for vaginal deliveries in hospitals. As a result, rural residents generally faced minimal out-of-pocket expenses.

## Methods

### Data Sources

This study used data from 2008 to 2020 from the National Maternal & Child Health Statistics (NMCHS) in China, a comprehensive database gathering maternal and child health records since the mid-1990s. Operating at the county level, the NMCHS encompasses both rural counties and urban districts (collectively referred to as *counties* for the purposes of this study). This dataset has been extensively used and cited in prior maternal and child health research in China for other topics, underscoring its significance and reliability.^15,19,20,21^ This study was a secondary data analysis at the aggregate county level, and no individual data were used, so it was exempt from human participants review by the internal review board at Peking University. Informed consent was not required. This study followed the Strengthening the Reporting of Observational Studies in Epidemiology (STROBE) reporting guideline.

### Variables and Rates

This study extracted data on the numbers of live births, hospital deliveries, and neonatal mortalities for all counties from 2008 to 2020. A total of 37 217 county-year records were included, while 268 county-year records were duplicated and 303 county-year records were excluded due to missing data on either hospital delivery or neonatal mortality (eFigure 1 in Supplement 1). Hospital deliveries at the county level were calculated as the percentage of live births occurring in hospitals. Hospital deliveries in China are defined as mothers giving birth in township, county, and city hospitals or higher-level medical facilities; all the hospitals and facilities should have maternal and neonatal care capacity. In contrast, the broader literature often includes under “facility deliveries” both hospitals with comprehensive services and clinics lacking such capacities. In China, although deliveries in clinics without maternal and neonatal service capacities have been technically prohibited since the strong promotion of hospital-based deliveries initiated in 2008, some newborns continued to be delivered in these clinics. Although these instances substantially decreased from 2008 to 2020, they are still captured in our dataset and categorized as nonhospital deliveries. Neonatal mortality was measured as the number of deaths within 28 days after delivery per 1000 live births at the county level. In addition, neonatal mortality at the provincial level was considered to ensure the completeness of neonatal mortality data at the county level, consistent with methods used in prior child mortality studies in China.^15,22^

### Statistical Analysis

Statistical analysis was conducted from March to December 2023 using Stata, version 17.0 (Stata Corp). Analysis was performed with *t* tests; all *P* values were from 2-sided tests and results were deemed statistically significant at *P* < .05. Previous studies conducted in China have shown that socioeconomic measures, such as gross domestic product (GDP) per capita and women’s education, are strongly correlated with child mortality.^15,16^ Moreover, local health resources, including the availability of hospital beds and health workers, were associated with health outcomes. Consequently, the present study included measures for GDP per capita and the mean years of education for females aged 15 to 49 years at the county level and the numbers of hospital beds and health workers per 1000 persons at the city level as control variables. These data were sourced from national, provincial, and city statistical yearbooks from 2008 to 2020 and censuses conducted in 2010 and 2020.^23,24^

To accurately assess the association between neonatal mortality and hospital delivery rates, this study applied multivariable fixed-effects linear models based on county-level cohort data. These models are designed to control for county-level characteristics that could be associated with neonatal mortality rates and remain constant over time. Such characteristics include, but are not limited to, health insurance coverage, established public health policies, and historical levels of health care access. Although these factors are consistent within a county across time, they can vary significantly between different counties. Furthermore, we performed stratified analyses based on urban or rural classification, geographical regions (western, central, and eastern), and GDP per capita quartiles.

We excluded 391 county-year records due to the absence of either socioeconomic or health resource control variables, resulting in 36 255 county-year records (97.4% of the initial dataset) being used for regression analyses (eFigure 1 in Supplement 1). For other instances of missing data in control variables, we used linear interpolation to estimate missing values based on available data across years.

The analysis dataset of NMCHS included a total of 198.7 million live births from 2930 counties across 36 646 county-year records in China from 2008 to 2020 (eTable 1 in Supplement 1). Both nationally and within the western, central, and eastern regions, rural areas accounted for a higher number of live births and encompassed more counties than their urban counterparts (eTables 2-4 in Supplement 1). The number of county-year records for each of the 31 provinces in mainland China is presented in eTable 5 in Supplement 1.

## Results

### Hospital Delivery and Neonatal Mortality Rates

Mean (SD) national hospital delivery rates demonstrated an increase from 94.7% (10.6%) in 2008 to 99.9% (0.5%) in 2020 (Table 1). By contrast, national neonatal mortality rates per 1000 live births decreased from a mean (SD) of 10.0 (7.3) in 2008 to 3.2 (2.5) in 2020. The mean (SD) neonatal mortality rate per 1000 live births decreased in rural areas from 12.3 (7.5) in 2008 to 3.9 (2.7) in 2020 and decreased in urban areas from 5.0 (3.1) in 2008 to 2.0 (1.3) in 2020. Hospital delivery rates increased in rural areas from a mean (SD) of 93.4% (11.8%) in 2008 to 99.9% (0.6%) in 2020 and increased in urban areas from a mean (SD) of 97.7% (6.1%) in 2008 to 100.0% (0.1%) in 2020. eTable 6 in Supplement 1 details the median (IQR) neonatal mortality rates and hospital delivery rates in 2008 and 2020. Consequently, the annual number of neonatal deaths in China decreased from 145 410 in 2008 to 37 810 in 2020. Hospital delivery rates experienced a rapid increase from 2008 to 2012 and subsequently sustained a very high level (eTable 7 in Supplement 1).

**Table 1.** Descriptive Statistics in 2008 and 2020^a^

Characteristic	2008			2020		
	All counties	Rural counties	Urban counties	All counties	Rural counties	Urban counties
No. of counties	2826	1965	861	2930	1994	936
No. of live births	14 483	10 017	4466	11 890	7455	4435
Neonatal mortality rate per 1000 live births, mean (SD)	10.0 (7.3)	12.3 (7.5)	5.0 (3.1)	3.2 (2.5)	3.9 (2.7)	2.0 (1.3)
Hospital delivery rate, mean (SD), %	94.7 (10.6)	93.4 (11.8)	97.7 (6.1)	99.9 (0.5)	99.9 (0.6)	100.0 (0.1)
GDP per capita (US $1000), mean (SD)	5.8 (11.9)	4.7 (11.8)	8.1 (11.8)	10.1 (7.4)	7.9 (5.6)	13.2 (8.5)
Women’s education, mean (SD), y	8.4 (1.6)	7.8 (1.2)	9.6 (1.5)	9.2 (1.5)	8.5 (1.0)	10.3 (1.3)
No. of hospital beds per 1000 persons, mean (SD)	3.0 (1.2)	2.8 (1.2)	3.2 (1.2)	5.3 (1.1)	5.2 (1.0)	5.4 (1.2)
No. of health workers per 1000 persons, mean (SD)	1.7 (0.7)	1.6 (0.6)	1.8 (0.7)	3.0 (0.9)	2.9 (0.8)	3.2 (0.9)

Abbreviation: GDP, gross domestic product.

^a^ Data on neonatal mortality and hospital delivery are from National Maternal & Child Health Statistics from 2008 to 2020 in China; data on GDP per capita, women’s education years, number of hospital beds per 1000 persons, and number of health workers per 1000 persons are from national, provincial, and city statistical yearbooks from 2008 to 2020 and censuses conducted in 2010 and 2020.

### Regional Trends in Hospital Delivery and Neonatal Mortality

In 2008, a disparity existed in hospital delivery rates between urban and rural areas in the western region, with mean (SD) rates of 84.6% (16.9%) in rural areas and 95.1% (9.5%) in urban areas. By 2020, these rates had significantly increased to 99.8% (1.0%) in rural areas and 100.0% (0.2%) in urban areas (Figure; eTable 8 in Supplement 1). All 3 regions achieved exceptionally high hospital delivery rates, averaging around 99% in both urban and rural areas (eTable 8 in Supplement 1). Between 2008 and 2020, rural areas across all 3 regions experienced larger reductions in neonatal mortality than their urban counterparts (Figure; eTable 9 in Supplement 1).

**Figure.**
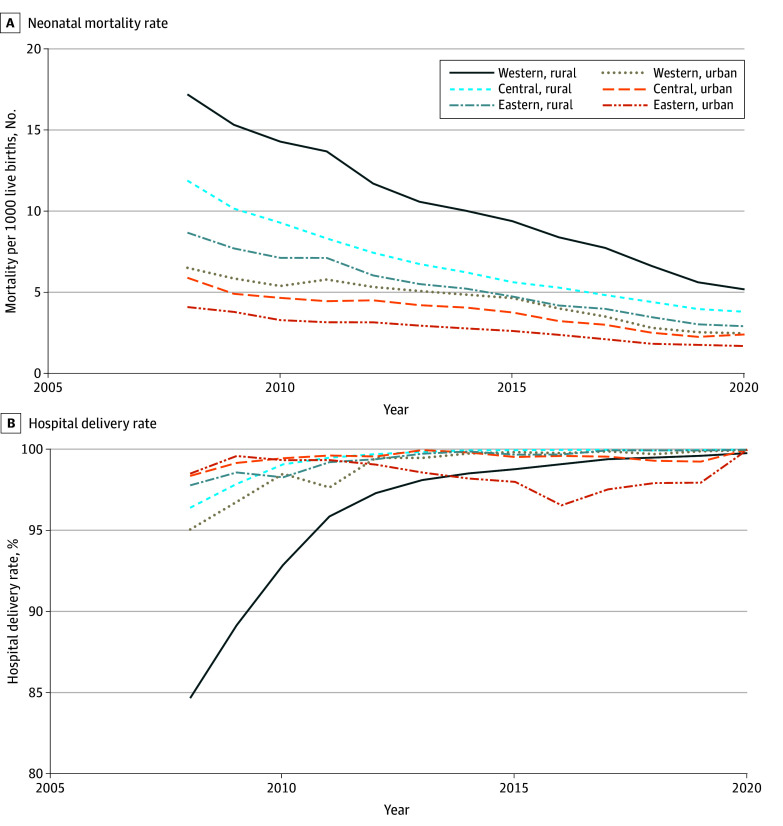
Trends of Neonatal Mortality and Hospital Delivery in China by Rural or Urban Status and 3 Regions in 2008-2020

In 2008, moderate heterogeneity was observed in hospital delivery rates at the county level (Figure), with the western region displaying a lower proportion of live births delivered in hospitals compared with those in the central and eastern regions. By 2020, hospital delivery rates across all 3 regions had increased and become uniformly high, effectively eliminating the disparities observed in previous years. Likewise, the regional heterogeneity of neonatal mortality rates across China decreased substantially from 2008 to 2020. In 2008, the rural areas in the western region recorded the lowest percentage of counties (28.9%) meeting the SDG 3.2.2 target of fewer than or equal to 12 neonatal deaths per 1000 live births. By 2020, this percentage had increased to 90.2% (eTable 10 in Supplement 1).

### Association Between Neonatal Mortality and Hospital Delivery by Rural and Urban Areas

Multivariable fixed-effects linear regression revealed a negative association between neonatal mortality and hospital delivery after adjusting for GDP per capita, women’s education years, and the number of hospital beds and health workers per 1000 persons. When we included all counties in the cohort dataset from 2008 to 2020 into the estimation (Table 2), the coefficient for hospital delivery was −10.08 (95% CI, −13.03 to −7.14; *P* < .001). This implies that an increase of 10 percentage points in hospital delivery was associated with a reduction of 1.008 neonatal deaths per 1000 live births. When rural and urban counties were analyzed separately, the coefficient for hospital delivery in rural areas was −14.16 (95% CI, −18.71 to −9.62; *P* < .001) compared with −0.72 (95% CI, −1.82 to 0.38; *P* = .20) in urban areas. In rural areas, an increase of 10 percentage points in hospital delivery was associated with a neonatal mortality rate of −1.42 (95% CI, −1.87 to −0.96; *P* < .001) per 1000 live births, whereas this negative association was not observed in urban areas. These findings highlight the significant association of hospital delivery with neonatal mortality in rural settings but not urban areas.

**Table 2.** Association of Neonatal Mortality and Hospital Delivery by Fixed-Effects Multivariable Estimation^a^

Variable	All (36 255 county-year records)		Rural (25 529 county-year records)		Urban (10 726 county-year records)	
	Coefficient (95% CI)	*P* value	Coefficient (95% CI)	*P* value	Coefficient (95% CI)	*P* value
Hospital delivery^b^	−10.08 (−13.03 to −7.14)	<.001	−14.16 (−18.71 to −9.62)	<.001	−0.72 (−1.82 to 0.38)	.20
GDP per capita (US $1000, in natural logarithm)	−1.74 (−2.04 to −1.44)	<.001	−2.40 (−2.87 to −1.92)	<.001	−0.53 (−0.72 to −0.33)	<.001
Women’s education, y	−0.74 (−1.22 to −0.26)	.002	−0.62 (−1.25 to 0.19)	.06	−0.79 (−1.05 to −0.53)	<.001
No. of hospital beds per 1000 population	−0.79 (−0.96 to −0.63)	<.001	−0.79 (−1.01 to −0.57)	<.001	−0.45 (−0.58 to −0.32)	<.001
No. of health workers per 1000 population	−0.85 (−1.05 to −0.66)	<.001	−1.11 (−1.38 to −0.84)	<.001	−0.45 (−0.65 to −0.25)	<.001
Constant	30.63 (26.40 to 34.85)	<.001	35.51 (30.03 to 40.98)	<.001	16.08 (13.31 to 18.85)	<.001

Abbreviation: GDP, gross domestic product.

^a^ Fixed-effects estimation of county-level cohort data between 2008 and 2020.

^b^ Measured between 0 and 1, indicating 0% to 100% of pregnant women who delivered newborns in hospitals.

The association between neonatal mortality and hospital delivery also showed regional variations in China. In the western region, the coefficients were −13.30 (95% CI, −17.51 to −9.08; *P* < .001) in rural areas and –1.03 (95% CI, −7.89 to 5.83; *P* = .77) in urban areas (Table 3). In the central region, this association was significant, with a coefficient of −25.34 (95% CI, −38.20 to −12.48; *P* < .001) in rural areas but a coefficient of only −0.66 (95% CI, −4.28 to 2.96; *P* = .72) in urban areas. By contrast, this association was not statistically significant in the eastern region, both for counties in rural areas (−2.35; 95% CI, −6.58 to 1.87; *P* = .27) and for counties in urban areas (−0.33; 95% CI, −0.87 to 0.21; *P* = .23).

**Table 3.** Association of Neonatal Mortality and Hospital Delivery by Fixed-Effects Multivariable Estimation in 3 Regions^a^

Characteristic	Western region						Central region						Eastern region					
	All^b^		Rural		Urban		All^b^		Rural		Urban		All^b^		Rural		Urban	
	Coefficient (95% CI)	*P* value	Coefficient (95% CI)	*P* value	Coefficient (95% CI)	*P* value	Coefficient (95% CI)	*P* value	Coefficient (95% CI)	*P* value	Coefficient (95% CI)	*P* value	Coefficient (95% CI)	*P* value	Coefficient (95% CI)	*P* value	Coefficient (95% CI)	*P* value
Sample size (county-year records)	13 832	NA	11 054	NA	2778	NA	11 391	NA	7920	NA	3471	NA	11 032	NA	6555	NA	4477	NA
Hospital delivery^c^	−13.48 (−17.48 to −9.48)	<.001	−13.30 (−17.51 to −9.08)	<.001	−1.03 (−7.89 to 5.83)	.77	−18.96 (−28.54 to −9.37)	<.001	−25.34 (−38.20 to −12.48)	<.001	−0.66 (−4.28 to 2.96)	.72	−1.45 (−2.72 to −0.19)	.02	−2.35 (−6.58 to 1.87)	.27	−0.33 (−0.87 to 0.21)	.23

Abbreviation: NA, not applicable.

^a^ Fixed-effects estimation of county-level cohort data between 2008 and 2020. Estimation also controls for gross domestic product per capita in natural logarithm, women’s education years, hospital beds per 1000 population, and health workers per 1000 population, and their coefficients are omitted.

^b^ Includes both rural and urban areas in the western, central, or eastern region.

^c^ Measured between 0 and 1, indicating 0% to 100% of pregnant women who delivered newborns in hospitals.

### Association Between Neonatal Mortality and Hospital Delivery by GDP per Capita Quartiles

All 2930 counties in 2020 were categorized into quartiles based on their GDP per capita. These quartiles were then applied retroactively, assigning the same counties from 2008 to 2019 to the quartiles determined by their 2020 GDP per capita rankings (eTable 11 in Supplement 1). Counties in the lowest quartile (<US $4854 in 2020) experienced the most substantial reduction in neonatal mortality rate, decreasing from a mean (SD) of 13.5 (7.9) per 1000 live births in 2008 to 4.1 (2.9) in 2020 (eFigure 2 and eTable 12 in Supplement 1). By contrast, counties in the highest quartile (>US $10 494 in 2020) observed the smallest reduction in neonatal mortality rate, decreasing from a mean (SD) of 6.0 (4.6) per 1000 live births in 2008 to 2.3 (1.7) in 2020. Likewise, counties in the lowest quartile experienced the largest increase in the hospital delivery rate, from a mean (SD) of 90.1% (14.2%) in 2008 to 100.0% (0.7%) among the quartiles of GDP per capita (eFigure 2 and eTable 13 in Supplement 1).

Multivariable fixed-effects linear analyses were conducted on the cohort data from 2008 to 2020 for each GDP per capita quartile separately (Table 4). In counties within the lowest GDP per capita quartile, the association between hospital delivery and neonatal mortality was strongly negative, with a coefficient of −14.00 (95% CI, −19.66 to −8.33; *P* < .001). By contrast, in the fourth quartile with the highest GDP per capita, no significant association between hospital delivery and neonatal mortalities was observed, with a coefficient of −0.68 (95% CI, −1.79 to 0.43; *P* = .23).

**Table 4.** Association of Neonatal Mortality and Hospital Delivery by Fixed-Effects Multivariable Estimation in GDP per Capita Quartiles^a^

GDP per capita in 2020	Quartile 1 (<US $4854)		Quartile 2 (US $4854-$6912)		Quartile 3 (US $6913-$10 494)		Quartile 4 (>US $10 494)	
	Coefficient (95% CI)	*P* value	Coefficient (95% CI)	*P* value	Coefficient (95% CI)	*P* value	Coefficient (95% CI)	*P* value
Sample size (county-year records)	9141	NA	9125	NA	9046	NA	8943	NA
Hospital delivery^b^	−14.00 (−19.66 to −8.33)	<.001	−13.41 (−20.56 to −6.26)	<.001	−8.90 (−15.37 to −2.42)	.007	−0.68 (−1.79 to 0.43)	.23
GDP per capita (natural logarithm)	−2.22 (−2.99 to −1.45)	<.001	−1.99 (−2.56 to −1.41)	<.001	−1.64 (−2.13 to −1.14)	<.001	−1.31 (−1.65 to −0.98)	<.001
Women’s education years	−0.49 (−2.30 to 1.33)	.60	−0.77 (−1.48 to −0.07)	.03	−1.11 (−1.64 to −0.58)	<.001	−0.64 (−0.96 to −0.32)	<.001
No. of hospital beds	−0.94 (−1.41 to −0.48)	<.001	−0.93 (−1.18 to −0.68)	<.001	−0.66 (−0.86 to −0.45)	<.001	−0.23 (−0.40 to −0.07)	.002
No. of health workers	−1.31 (−1.89 to −0.73)	<.001	−1.04 (−1.39 to −0.69)	<.001	−0.66 (−0.96 to −0.36)	<.001	−0.63 (−0.89 to −0.38)	<.001
Constant	34.36 (22.95 to 45.76)	<.001	35.22 (27.54 to 42.89)	<.001	31.34 (23.22 to 39.46)	<.001	16.74 (13.79 to 19.78)	<.001

Abbreviations: GDP, gross domestic product; NA, not applicable.

^a^ Fixed-effects estimation of county-level cohort data between 2008 and 2020.

^b^ Measured between 0 and 1, indicating 0% to 100% of pregnant women who delivered newborns in hospitals.

## Discussion

The decrease in neonatal mortality rates across China from 2008 to 2020 represents a significant accomplishment. By 2020, more than 96% of the 2930 counties had achieved a neonatal mortality rate below the SDG 3.2.2 target of fewer than or equal to 12 per 1000 live births. China has maintained a hospital delivery rate of more than 99% at the county level since 2016. Our analyses demonstrate a consistently negative association between neonatal mortality and hospital delivery in rural China, while this association is not observed in urban areas. Furthermore, this negative association is particularly pronounced in the less-developed western and central regions of China, in contrast to the more developed eastern region. The study underscores this negative association primarily in counties with lower quartiles of GDP per capita, while it is absent in counties within the highest quartile of GDP per capita.

Previous studies have examined the association between neonatal mortality and hospital or facility delivery primarily in African and South Asian countries, with mixed results.^4,5,6,7,8,9,10,11,12,13,14,25,26,27,28,29,30,31^ In reviewing the association of conditional cash transfers with maternal and neonatal health outcomes, we found parallels and distinctions across global studies. A systematic review of interventions in 8 countries reported increased facility-based deliveries due to conditional cash transfers, although these studies lacked consistent documentation of their associations with maternal and newborn mortality.^25^ A conditional cash transfer program in India was associated with a significant reduction in neonatal mortality rate, decreasing by 2.3 deaths per 1000 live births.^26^ Similarly, in Brazil, a program targeting pregnant women effectively reduced the incidence of low birth weight by 11%, highlighting the potential of such interventions to influence critical early-life health outcomes.^27^ Moreover, cash transfer programs generally correlate with significant reductions in maternal mortality and mortality among childen younger than 5 years across low- and middle-income countries.^28^ Previous studies also showed that delivery in a higher-quality facility was associated with a 2.3–percentage point lower newborn mortality rate^29^; however, facility delivery with postnatal checkup was associated with reduced odds of neonatal death,^30^ and safe childbirth requires quality of delivery more than quality of hospitals or facilities.^31^ On the other hand, many studies showed no association between hospital delivery and a reduction in neonatal mortality across a broad range of economic development and urbanization levels in Ghana, India, Kenya, Malawi, and Zambia.^9,10,11,12,13,14^

Our findings from China, an upper-middle-income country, provide different evidence of a negative association between neonatal mortality and hospital delivery, especially in rural areas, in the western and central regions, and in counties with lower GDP per capita. Promoting hospital delivery in these settings could be associated with further decreases in neonatal mortality rates, providing valuable insights and policy implications for other low- and middle-income countries aiming to enhance maternal and neonatal health. Our study offers valuable insights into the associations of hospital delivery policies with neonatal outcomes within the Chinese context, yet recognizes that the direct applicability to other countries may be moderated by differences in health care systems, cultural factors, and policy environments.

Urban and economically developed regions with higher GDP per capita in China experienced a significant increase in the influx of internal migrants from rural and economically underdeveloped areas between 2008 and 2020. These migrants often have lower rates of hospital delivery compared with local residents, contributing to the observed variability in hospital delivery rates. Moreover, the nonsignificant association between hospital delivery and neonatal outcomes in these urban and economically developed regions could be explained by the already high baseline standard of maternal and neonatal health care, which may reduce the association of increased hospital deliveries with neonatal outcomes.

### Limitations

This study has several limitations. First, as with any observational study, our analysis may have been prone to unobserved confounding factors. To mitigate this, we used fixed-effects models on cohort data from more than 2900 counties from 2008 to 2020, aiming to control for time-invariant unobserved factors. However, potential time-varying unobserved confounders, such as economic fluctuations or policy changes that differ within a county over time, might still have influenced the observed trends. Second, the reliability of our findings depends on the quality of data from the NMCHS of China. Concerns about data quality include accuracy of local reporting and potential underreporting of neonatal mortality in rural areas. This dataset has been validated for its high quality through numerous publications in previous literature.^19,20,21^ Third, our findings may not be universally applicable, particularly in settings where structural and interpersonal factors significantly influence the safety and effectiveness of hospital deliveries. Disparities in health care access and outcomes can profoundly affect maternal and neonatal health and should be carefully considered when interpreting these results.

## Conclusions

In this cohort study of more than 2900 counties, our findings highlight that an increase in hospital deliveries was associated with reduced neonatal mortality in rural and economically underdeveloped areas in China. Hospital delivery remains a critical public health intervention, particularly in rural, less-developed areas, as well as in western and central regions of China. To further reduce neonatal mortality and improve newborn health, it is imperative to increase the accessibility of hospital delivery services. In addition, enhancing public health campaigns to inform and encourage expecting mothers to use hospital facilities for childbirth is crucial. For counties in the lower GDP quartiles, tailoring policy recommendations to include varied hospital delivery subsidies based on household incomes could prove more effective. Promoting hospital delivery could be associated with further decreases in neonatal mortality rates, providing valuable insights and policy implications for other low- and middle-income countries.

## Notes

Supplement 1. **eTable 1.** Counties and Live Births From 2008 to 2020 in China **eTable 2.** Counties and Live Births From 2008 to 2020 in the Western Region **eTable 3.** Counties and Live Births From 2008 to 2020 in the Central Region **eTable 4.** Counties and Live Births From 2008 to 2020 in the Eastern Region **eTable 5.** The Number of County-Year Records From 2008 to 2020 in All 31 Provinces in Mainland China **eTable 6.** Descriptive Statistics of Median and Interquartile Range in 2008 and 2020 **eTable 7.** Neonatal Mortality and Hospital Delivery From 2008 to 2020 in China **eTable 8.** Hospital Delivery Rate From 2008 to 2020 by Three Regions **eTable 9.** Neonatal Mortality Rate From 2008 to 2020 by Three Regions **eTable 10.** Counties With Neonatal Mortality Rate Less Than or Equal to 12 per 1000 Live Births From 2008 to 2020 by Three Regions **eTable 11.** Counties and Live Births by Four Quartiles of GDP per Capita **eTable 12.** Neonatal Mortality Rate From 2008 to 2020 by Four Quartiles of GDP per Capita **eTable 13.** Hospital Delivery Rate From 2008 to 2020 by Four Quartiles of GDP per Capita **eFigure 1.** County-Year Records Cleaning Flow Diagram **eFigure 2.** Trends of Neonatal Mortality and Hospital Delivery From 2008 to 2020 by Four Quartiles of GDP per Capita

Supplement 2.
Data Sharing Statement
