# Supplementary material for: Regional Disparities, Economic Development, and Neonatal Mortality and Hospital Delivery in China
Source: JAMA Netw Open. 2024 Nov 6;7(11):e2443423. doi: 10.1001/jamanetworkopen.2024.43423 (PMC11541646; doi:10.1001/jamanetworkopen.2024.43423)
Supplement: Supplement 1. — eTable 1. Counties and Live Births From 2008 to 2020 in China eTable 2. Counties and Live Births From 2008 to 2020 in the Western Region eTable 3. Counties and Live Births From 2008 to 2020 in the Central Region eTable 4. Counties and Live Births From 2008 to 2020 in the Eastern Region eTable 5. The Number of County-Year Records From 2008 to 2020 in All 31 Provinces in Mainland China eTable 6. Descriptive Statistics of Median and Interquartile Range in 2008 and 2020 eTable 7. Neonatal Mortality and Hospital Delivery From 2008 to 2020 in China eTable 8. Hospital Delivery Rate From 2008 to 2020 by Three Regions eTable 9. Neonatal Mortality Rate From 2008 to 2020 by Three Regions eTable 10. Counties With Neonatal Mortality Rate Less Than or Equal to 12 per 1000 Live Births From 2008 to 2020 by Three Regions eTable 11. Counties and Live Births by Four Quartiles of GDP per Capita eTable 12. Neonatal Mortality Rate From 2008 to 2020 by Four Quartiles of GDP per Capita eTable 13. Hospital Delivery Rate From 2008 to 2020 by Four Quartiles of GDP per Capita eFigure 1. County-Year Records Cleaning Flow Diagram eFigure 2. Trends of Neonatal Mortality and Hospital Delivery From 2008 to 2020 by Four Quartiles of GDP per Capita [file jamanetwopen-e2443423-s001.pdf]

Supplementary Online Content

Fang H, Zhang H, Vargas Bustamante A, et al. Regional disparities, economic development, and neonatal mortality and hospital delivery in China. *JAMA Netw Open.* 2024;7(11):e2443423. doi:10.1001/jamanetworkopen.2024.43423

eTable 1. Counties and Live Births From 2008 to 2020 in China
eTable 2. Counties and Live Births From 2008 to 2020 in the Western Region
eTable 3. Counties and Live Births From 2008 to 2020 in the Central Region
eTable 4. Counties and Live Births From 2008 to 2020 in the Eastern Region
eTable 5. The Number of County-Year Records From 2008 to 2020 in All 31 Provinces in Mainland China
eTable 6. Descriptive Statistics of Median and Interquartile Range in 2008 and 2020
eTable 7. Neonatal Mortality and Hospital Delivery From 2008 to 2020 in China
eTable 8. Hospital Delivery Rate From 2008 to 2020 by Three Regions
eTable 9. Neonatal Mortality Rate From 2008 to 2020 by Three Regions
eTable 10. Counties With Neonatal Mortality Rate Less Than or Equal to 12 per 1000 Live Births From 2008 to 2020 by Three Regions
eTable 11. Counties and Live Births by Four Quartiles of GDP per Capita
eTable 12. Neonatal Mortality Rate From 2008 to 2020 by Four Quartiles of GDP per Capita
eTable 13. Hospital Delivery Rate From 2008 to 2020 by Four Quartiles of GDP per Capita
eFigure 1. County-Year Records Cleaning Flow Diagram
eFigure 2. Trends of Neonatal Mortality and Hospital Delivery From 2008 to 2020 by Four Quartiles of GDP per Capita

This supplementary material has been provided by the authors to give readers additional information about their work.

© 2024 Fang H et al. *JAMA Network Open.*

eTable 1: Counties and Live Births From 2008 to 2020 in China

Year	All		Rural		Urban	
	Counties	Live Births (million)	Counties	Live Births (million)	Counties	Live Births (million)
2008	2,826	14.49	1,965	10.02	861	4.47
2009	2,822	15.02	1,966	10.44	856	4.58
2010	2,766	15.03	1,973	10.82	793	4.21
2011	2,790	15.57	1,974	11.04	816	4.53
2012	2,792	16.60	1,974	11.63	818	4.97
2013	2,793	16.24	1,974	11.42	819	4.82
2014	2,822	16.42	1,975	11.31	847	5.11
2015	2,819	15.69	1,973	10.76	846	4.93
2016	2,820	15.98	1,973	10.75	847	5.23
2017	2,822	17.48	1,974	11.54	848	5.94
2018	2,822	14.52	1,974	9.45	848	5.07
2019	2,822	13.74	1,974	8.72	848	5.02
2020	2,930	11.89	1,994	7.45	936	4.44
Total	36,646	198.67	25,663	135.35	10,983	63.32

© 2024 Fang H et al. *JAMA Network Open.*

eTable 2: Counties and Live Births From 2008 to 2020 in the Western Region

Year	Western		Western, Rural		Western, Urban	
	Counties	Live Births (million)	Counties	Live Births (million)	Counties	Live Births (million)
2008	1,060	4.00	851	2.96	209	1.04
2009	1,054	4.14	851	3.07	203	1.07
2010	1,063	4.25	851	3.15	212	1.10
2011	1,059	4.25	851	3.18	208	1.07
2012	1,061	4.48	851	3.30	210	1.18
2013	1,061	4.46	851	3.29	210	1.17
2014	1,075	4.44	852	3.25	223	1.19
2015	1,073	4.30	851	3.17	222	1.13
2016	1,073	4.36	851	3.16	222	1.20
2017	1,074	4.81	852	3.42	222	1.39
2018	1,074	4.22	852	2.94	222	1.28
2019	1,074	4.08	852	2.78	222	1.30
2020	1,090	3.64	861	2.50	229	1.14
Total	13,891	55.43	11,077	40.17	2,814	15.26

© 2024 Fang H et al. *JAMA Network Open.*

eTable 3: Counties and Live Births From 2008 to 2020 in the Central Region

Year	Central		Central, Rural		Central, Urban	
	Counties	Live Births (million)	Counties	Live Births (million)	Counties	Live Births (million)
2008	887	4.51	605	3.52	282	0.99
2009	891	4.71	606	3.68	285	1.03
2010	857	4.82	610	3.91	247	0.91
2011	878	5.02	611	4.00	267	1.02
2012	878	5.35	611	4.21	267	1.14
2013	879	5.24	611	4.13	268	1.11
2014	885	5.19	611	4.01	274	1.18
2015	886	4.96	611	3.81	275	1.15
2016	886	4.99	611	3.78	275	1.21
2017	886	5.21	611	3.90	275	1.31
2018	885	4.36	611	3.24	274	1.12
2019	885	4.04	611	2.95	274	1.09
2020	928	3.39	618	2.47	310	0.92
Total	11,511	61.79	7,938	47.61	3,573	14.18

© 2024 Fang H et al. *JAMA Network Open.*

eTable 4: Counties and Live Births From 2008 to 2020 in the Eastern Region

Year	Eastern		Eastern, Rural		Eastern, Urban	
	Counties	Live Births (million)	Counties	Live Births (million)	Counties	Live Births (million)
2008	879	5.97	509	3.54	370	2.43
2009	877	6.17	509	3.69	368	2.48
2010	846	5.96	512	3.76	334	2.20
2011	853	6.29	512	3.86	341	2.43
2012	853	6.77	512	4.12	341	2.65
2013	853	6.55	512	4.00	341	2.55
2014	862	6.79	512	4.05	350	2.74
2015	860	6.43	511	3.78	349	2.65
2016	861	6.63	511	3.81	350	2.82
2017	862	7.47	511	4.23	351	3.24
2018	863	5.94	511	3.27	352	2.67
2019	863	5.63	511	2.99	352	2.64
2020	912	4.85	515	2.48	397	2.37
Total	11,244	81.45	6,648	47.58	4,596	33.87

© 2024 Fang H et al. *JAMA Network Open.*

eTable 5. The Number of County-Year Records From 2008 to 2020 in All 31 Provinces in Mainland China

Province	Number of County-Year Records	Percent (%)
**Eastern Region**		
Beijing	208	0.57
Tianjin	200	0.55
Hebei	2,213	6.04
Liaoning	1,322	3.61
Shanghai	208	0.57
Jiangsu	1,211	3.30
Zhejiang	1,166	3.18
Fujian	1,095	2.99
Shandong	1,805	4.93
Guangdong	1,575	4.30
Hainan	241	0.66
**Central Region**		
Shanxi	1,519	4.15
Jilin	795	2.17
Heilongjiang	1,583	4.32
Anhui	1,366	3.73
Jiangxi	1,312	3.58
Henan	2,046	5.58
Hubei	1,302	3.55
Hunan	1,588	4.33
**Western Region**		
Inner Mongolia	1,321	3.60
Guangxi	1,397	3.81
Chongqing	503	1.37
Sichuan	2,367	6.46
Guizhou	1,132	3.09
Yunnan	1,677	4.58
Xizang	952	2.60
Shaanxi	1,392	3.80
Gansu	1,121	3.06
Qinghai	539	1.47
Ningxia	287	0.78
Xinjiang	1,203	3.28
**Total**	**36,646**	**100.00**

© 2024 Fang H et al. *JAMA Network Open.*

eTable 6: Descriptive Statistics of Median and Interquartile Range in 2008 and 2020

	2008			2020		
	All	Rural	Urban	All	Rural	Urban
Number of counties	2,826	1,965	861	2,930	1,994	936
Number of live births (thousand)	14,483	10,017	4,466	11,890	7,455	4,435
	**Median (IQR)**	**Median (IRQ)**	**Median (IQR)**	**Median (IQR)**	**Median (IQR)**	**Median (IQR)**
Neonatal mortality (per 1,000 live births)	8,12 (8.75)	10.42 (8.71)	4.37 (3.11)	2.63 (2.65)	3.38 (2.74)	1.67 (1.49)
Hospital delivery (%)	99.49 (5.45)	98.75 (7.56)	99.93 (1.02)	100.00 (0.01)	100.00 (0.02)	100.00 (0.00)
GDP per capita (USD 1,000)	3.16 (4.45)	2.50 (2.98)	5.41 (6.42)	7.80 (7.28)	6.07 (4.75)	11.07 (8.39)
Women's education years	7.98 (1.23)	7.92 (1.15)	9.81 (2.14)	8.87 (2.09)	8.31 (0.99)	10.40 (1.83)
Number of hospital beds (per 1,000 persons)	2.75 (1.10)	2.62 (1.04)	3.12 (1.47)	5.28 (1.53)	5.22 (1.33)	5.45 (1.70)
Number of health workers (per 1,000 persons)	1.53 (0.71)	1.43 (0.65)	1.79 (1.00)	2.97 (0.87)	2.87 (0.88)	3.22 (0.89)

Data source: data of neonatal mortality and hospital delivery are from National Maternal & Child Health Statistics (NMCHS) 2008-2020 in China; data of GDP per capita, women's education years, number of hospital beds per 1,000 persons, and number of health workers per 1,000 persons are from national, provincial, and city statistical yearbooks in 2008-2020, and censuses conducted in 2010 and 2020.
IQR: interquartile range

© 2024 Fang H et al. *JAMA Network Open.*

eTable 7: Neonatal Mortality and Hospital Delivery From 2008 to 2020 in China

Year	Neonatal Mortality Rate (per 1,000 livebirths)			Hospital Delivery Rate (%)		
	All	Rural	Urban	All	Rural	Urban
	Mean (SD)	Mean (SD)	Mean (SD)	Mean (SD)	Mean (SD)	Mean (SD)
2008	10.04 (7.31)	12.28 (7.52)	4.99 (3.13)	94.72 (10.56)	93.41 (11.79)	97.67 (6.10)
2009	8.85 (6.48)	10.77 (6.74)	4.46 (2.59)	96.55 (8.26)	95.55 (9.32)	98.84 (4.24)
2010	8.30 (6.72)	9.95 (7.12)	4.07 (2.50)	97.59 (7.48)	96.99 (8.29)	99.15 (4.44)
2011	7.83 (6.08)	9.40 (6.39)	4.01 (2.58)	98.54 (6.11)	98.35 (5.75)	99.01 (6.89)
2012	6.85 (5.20)	8.10 (5.53)	3.91 (2.55)	99.03 (4.77)	98.92 (4.63)	99.28 (5.09)
2013	6.27 (4.66)	7.36 (4.97)	3.69 (2.24)	99.25 (4.29)	99.31 (3.25)	99.11 (6.08)
2014	5.84 (4.33)	6.91 (4.64)	3.49 (2.12)	99.34 (5.01)	99.52 (2.70)	98.94 (8.03)
2015	5.40 (4.17)	6.37 (4.55)	3.28 (1.91)	99.28 (5.67)	99.52 (3.44)	98.78 (8.74)
2016	4.81 (3.74)	5.75 (4.09)	2.88 (1.67)	99.09 (7.11)	99.62 (3.21)	98.00 (11.47)
2017	4.38 (3.40)	5.32 (3.72)	2.56 (1.50)	99.37 (5.50)	99.80 (1.36)	98.53 (9.18)
2018	3.82 (3.14)	4.71 (3.47)	2.16 (1.26)	99.41 (5.32)	99.81 (1.39)	98.68 (8.75)
2019	3.24 (2.68)	4.11 (2.97)	2.00 (1.26)	99.44 (5.47)	99.85 (1.19)	98.73 (8.87)
2020	3.18 (2.47)	3.90 (2.69)	1.97 (1.32)	99.94 (0.47)	99.92 (0.59)	99.98 (0.11)

© 2024 Fang H et al. *JAMA Network Open.*

eTable 8: Hospital Delivery Rate From 2008 to 2020 by Three Regions

Yeas	Hospital delivery rate (%)								
	Western			Central			Eastern		
	All	Rural	Urban	All	Rural	Urban	All	Rural	Urban
	Mean (SD)	Mean (SD)	Mean (SD)	Mean (SD)	Mean (SD)	Mean (SD)	Mean (SD)	Mean (SD)	Mean (SD)
2008	87.35 (15.99)	84.63 (16.91)	95.05 (9.46)	96.83 (5.42)	96.40 (5.59)	98.37 (4.39)	98.08 (5.08)	97.78 (5.55)	98.51 (4.26)
2009	91.10 (13.23)	89.13 (14.15)	96.76 (7.65)	98.15 (3.63)	97.87 (3.82)	99.17 (2.59)	98.99 (3.27)	98.58 (3.95)	99.60 (1.66)
2010	94.32 (10.29)	92.86 (11.34)	98.50 (4.25)	99.15 (1.65)	99.08 (1.67)	99.46 (1.56)	98.67 (7.24)	98.28 (8.15)	99.34 (5.25)
2011	96.31 (9.66)	95.86 (8.88)	97.65 (11.57)	99.52 (1.18)	99.49 (1.14)	99.63 (1.33)	99.27 (4.86)	99.22 (4.59)	99.35 (5.25)
2012	97.88 (6.21)	97.30 (7.00)	99.49 (2.45)	99.69 (1.30)	99.72 (0.94)	99.57 (2.17)	99.27 (5.27)	99.40 (4.17)	99.07 (6.62)
2013	98.47 (5.17)	98.11 (5.52)	99.48 (3.86)	99.87 (0.44)	99.85 (0.49)	99.96 (0.15)	99.29 (5.16)	99.74 (1.79)	98.58 (7.91)
2014	98.85 (4.27)	98.52 (4.79)	99.75 (2.05)	99.92 (0.89)	99.95 (0.15)	99.82 (1.85)	99.21 (6.92)	99.88 (0.88)	98.21 (10.76)
2015	99.06 (3.41)	98.78 (3.89)	99.84 (1.02)	99.86 (1.84)	99.96 (0.13)	99.54 (3.79)	98.99 (8.23)	99.68 (4.50)	98.00 (11.58)
2016	99.28 (2.87)	99.09 (3.17)	99.78 (1.74)	99.89 (1.43)	99.98 (0.08)	99.60 (2.89)	98.36 (10.68)	99.69 (4.52)	96.55 (15.32)
2017	99.54 (2.03)	99.40 (2.32)	99.89 (0.98)	99.87 (1.49)	99.98 (0.14)	99.56 (2.93)	98.90 (8.13)	99.95 (0.70)	97.54 (12.19)
2018	99.57 (2.20)	99.50 (2.20)	99.71 (2.19)	99.78 (2.12)	99.94 (0.36)	99.31 (4.10)	99.04 (7.88)	99.95 (1.00)	97.93 (11.60)
2019	99.70 (1.50)	99.61 (1.73)	99.89 (0.75)	99.78 (3.05)	99.97 (0.43)	99.25 (5.80)	99.01 (8.03)	99.94 (1.03)	97.94 (11.58)
2020	99.83 (0.84)	99.78 (1.00)	99.95 (0.20)	99.99 (0.04)	99.99 (0.04)	99.99 (0.04)	99.98 (0.07)	99.98 (0.07)	99.98 (0.06)

© 2024 Fang H et al. *JAMA Network Open.*

eTable 9: Neonatal Mortality Rate From 2008 to 2020 by Three Regions

Year	Neonatal mortality rate (per 1000 livebirths)								
	Western			Central			Eastern		
	All	Rural	Urban	All	Rural	Urban	All	Rural	Urban
	Mean (SD)	Mean (SD)	Mean (SD)	Mean (SD)	Mean (SD)	Mean (SD)	Mean (SD)	Mean (SD)	Mean (SD)
2008	14.38 (9.44)	17.18 (9.26)	6.45 (3.69)	10.53 (5.35)	11.85 (5.18)	5.84 (2.60)	6.75 (4.97)	8.63 (5.30)	4.03 (2.69)
2009	12.85 (8.32)	15.30 (8.19)	5.79 (2.90)	8.96 (5.06)	10.11 (5.00)	4.85 (2.45)	6.08 (4.23)	7.65 (4.52)	3.73 (2.21)
2010	11.94 (7.96)	14.26 (7.89)	5.33 (2.81)	8.36 (6.66)	9.24 (7.00)	4.59 (2.58)	5.65 (4.04)	7.07 (4.27)	3.22 (1.90)
2011	11.66 (7.99)	13.65 (8.17)	5.73 (2.93)	7.48 (4.49)	8.27 (4.52)	4.39 (2.66)	5.53 (4.10)	7.07 (4.37)	3.09 (1.85)
2012	9.98 (7.02)	11.66 (7.25)	5.27 (3.19)	6.76 (3.78)	7.39 (3.83)	4.44 (2.48)	4.85 (3.44)	5.98 (3.74)	3.08 (1.84)
2013	9.09 (6.18)	10.54 (6.46)	5.02 (2.44)	6.14 (3.34)	6.69 (3.38)	4.14 (2.24)	4.45 (3.19)	5.45 (3.48)	2.88 (1.74)
2014	8.59 (5.87)	9.98 (6.15)	4.80 (2.42)	5.68 (2.96)	6.18 (2.98)	3.99 (2.15)	4.17 (2.88)	5.17 (3.14)	2.71 (1.54)
2015	8.09 (5.76)	9.34 (6.13)	4.59 (2.09)	5.13 (2.83)	5.57 (2.90)	3.69 (1.98)	3.80 (2.58)	4.68 (2.85)	2.55 (1.38)
2016	7.13 (5.07)	8.34 (5.37)	3.94 (1.89)	4.73 (2.88)	5.23 (2.99)	3.16 (1.72)	3.35 (2.19)	4.12 (2.41)	2.31 (1.25)
2017	6.46 (4.79)	7.68 (5.08)	3.44 (1.76)	4.30 (2.49)	4.77 (2.56)	2.93 (1.61)	3.10 (1.89)	3.91 (1.99)	2.04 (1.04)
2018	5.41 (4.44)	6.57 (4.79)	2.74 (1.43)	3.86 (2.29)	4.34 (2.35)	2.44 (1.36)	2.66 (1.75)	3.40 (1.91)	1.76 (0.94)
2019	4.58 (3.66)	5.56 (3.97)	2.47 (1.37)	3.44 (2.11)	3.90 (2.18)	2.19 (1.20)	2.36 (1.61)	2.96 (1.73)	1.69 (1.14)
2020	4.27 (3.19)	5.12 (3.39)	2.40 (1.49)	3.36 (2.15)	3.74 (2.21)	2.33 (1.59)	2.24 (1.49)	2.84 (1.64)	1.62 (0.97)

© 2024 Fang H et al. *JAMA Network Open.*

eTable 10: Counties With Neonatal Mortality Rate Less Than or Equal To 12 per 1000 Live Births From 2008 to 2020 by Three Regions

Year	West				Central				East			
	Rural		Urban		Rural		Urban		Rural		Urban	
	Counties	NMR <=12	Counties	NMR <=12	Counties	NMR<=12	Counties	NMR <=12	Counties	NMR <=12	Counties	NMR <=12
2008	851	28.91%	209	90.91%	605	56.69%	282	94.68%	509	77.21%	370	97.57%
2009	851	33.73%	203	93.60%	606	65.85%	285	97.89%	509	83.69%	368	98.10%
2010	851	39.13%	212	94.34%	610	70.98%	247	96.76%	512	86.33%	334	99.10%
2011	851	41.95%	208	94.23%	611	74.14%	267	95.51%	512	85.74%	341	99.41%
2012	851	53.82%	210	93.33%	611	81.67%	267	97.38%	512	90.04%	341	99.41%
2013	851	58.75%	210	95.71%	611	86.74%	268	97.76%	512	94.53%	341	99.71%
2014	852	63.73%	223	95.96%	611	91.49%	274	99.27%	512	96.69%	350	99.43%
2015	851	67.80%	222	98.65%	611	91.33%	275	99.27%	511	96.67%	349	99.43%
2016	851	76.50%	222	98.20%	611	93.94%	275	98.91%	511	98.04%	350	100.00%
2017	852	77.46%	222	98.65%	611	95.42%	275	98.91%	511	99.41%	351	99.72%
2018	852	81.46%	222	99.55%	611	97.22%	274	99.27%	511	99.61%	352	99/72%
2019	852	86.27%	222	99.55%	611	97.38%	274	98.54%	511	99.61%	352	99.43%
2020	861	90.24%	226	98.69%	599	96.93%	304	98.06%	515	99.61%	397	100.00%
Total	11,077		2,399		7,919		3,567		6,648		4,596	

Note: Neonatal mortality rate (NMR)

© 2024 Fang H et al. *JAMA Network Open.*

eTable 11: Counties and Live Births by Four Quartiles of GDP per Capita

GDP per capita[1]	Quartile 1 (Less than USD 4,854)		Quartile 2 (USD 4,854 – 6,912)		Quartile 3 (USD 6,913 – 10,494)		Quartile 4 (More than USD 10,494)	
**Year**	**Counties**	**Livebirths (million)**	**Counties**	**Livebirths (million)**	**Counties**	**Livebirths (million)**	**Counties**	**Livebirths (million)**
2008	701	3.78	700	3.41	680	3.19	657	3.53
2009	701	4.03	700	3.59	675	3.26	657	3.53
2010	702	4.17	706	3.73	691	3.45	667	3.67
2011	704	4.26	707	3.79	694	3.54	685	3.97
2012	704	4.48	708	4.00	695	3.75	685	4.37
2013	704	4.45	709	3.89	693	3.61	687	4.29
2014	706	4.38	710	3.86	707	3.71	699	4.47
2015	707	4.14	706	3.65	707	3.56	699	4.34
2016	707	4.05	706	3.70	707	3.67	700	4.56
2017	707	4.23	706	4.00	707	4.14	702	5.11
2018	707	3.47	707	3.34	705	3.42	703	4.29
2019	707	3.21	706	3.10	706	3.25	703	4.18
2020	706	2.81	707	2.67	707	2.70	707	3.36
Total	9,163	51.46	9,178	46.73	9,074	45.25	8,951	53.67

1. GDP per capita in the year of 2020.

© 2024 Fang H et al. *JAMA Network Open.*

eTable 12: Neonatal Mortality Rate From 2008 to 2020 by Four Quartiles of GDP per Capita

Year	Neonatal mortality rate (per 1000 livebirths)			
GDP per capita[1]	Quartile 1 (less than USD 4854)	Quartile 2 (USD 4854 - 6912)	Quartile3 (USD 6913 - 10494)	Quartile 4 (more than USD 10494)
	Mean (SD)	Mean (SD)	Mean (SD)	Mean (SD)
2008	13.47 (7.89)	12.27 (7.29)	9.01 (6.62)	6.01 (4.60)
2009	12.00 (7.32)	10.51 (6.25)	7.88 (5.57)	5.27 (4.12)
2010	11.34 (8.61)	9.36 (5.84)	7.19 (5.29)	4.83 (3.87)
2011	10.58 (7.21)	8.85 (5.79)	6.87 (4.97)	4.76 (3.95)
2012	9.06 (6.14)	7.71 (5.14)	6.08 (4.39)	4.45 (3.39)
2013	8.31 (5.63)	6.89 (4.35)	5.68 (4.06)	4.09 (2.93)
2014	7.70 (5.32)	6.48 (4.12)	5.28 (3.70)	3.95 (2.74)
2015	7.19 (5.18)	5.98 (4.13)	4.78 (3.49)	3.71 (2.54)
2016	6.37 (4.84)	5.42 (3.67)	4.44 (3.06)	3.25 (2.17)
2017	5.81 (4.36)	5.03 (3.51)	4.10 (2.71)	2.93 (2.02)
2018	5.18 (4.29)	4.41 (2.96)	3.48 (2.43)	2.53 (1.85)
2019	4.40 (3.40)	3.94 (2.76)	3.09 (2.19)	2.26 (1.76)
2020	4.14 (2.94)	3.66 (2.63)	3.00 (2.11)	2.30 (1.74)

1. GDP per capita in the year of 2020.

© 2024 Fang H et al. *JAMA Network Open.*

eTable 13: Hospital Delivery Rate From 2008 to 2020 by Four Quartiles of GDP per Capita

Year	Hospital delivery rate (%)			
GDP per capita[1]	Quartile 1 (less than USD 4854)	Quartile 2 (USD 4854 - 6912)	Quartile3 (USD 6913 - 10494)	Quartile 4 (more than USD 10494)
	Mean (SD)	Mean (SD)	Mean (SD)	Mean (SD)
2008	90.09 (14.15)	92.60(11.44)	97.33 (6.91)	99.12 (4.02)
2009	93.29 (11.71)	95.25 (8.71)	98.28 (4.96)	99.50 (2.56)
2010	95.77 (9.29)	97.35 (5.78)	98.99 (3.52)	98.60 (8.86)
2011	97.46 (7.19)	98.60 (3.91)	99.21 (4.70)	99.05 (7.39)
2012	98.34 (5.69)	99.15 (2.92)	99.58 (2.17)	99.15 (6.38)
2013	98.83 (4.55)	99.46 (2.22)	99.57 (3.57)	99.23 (5.73)
2014	99.18 (3.85)	99.64 (1.68)	99.49 (4.20)	99.10 (7.78)
2015	99.31 (3.09)	99.66 (2.26)	99.21 (6.57)	98.99 (8.21)
2016	99.48 (2.57)	99.70 (2.18)	99.24 (6.59)	98.12 (11.45)
2017	99.64 (1.97)	99.67 (3.43)	99.50 (3.99)	98.79 (8.81)
2018	99.66 (1.95)	99.68 (3.50)	99.45 (4.08)	98.98 (8.33)
2019	99.77 (1.47)	99.70 (3.60)	99.45 (4.69)	98.98 (8.34)
2020	99.87 (0.68)	99.92 (0.56)	99.97 (0.42)	99.98 (0.07)

1. GDP per capita in the year of 2020.

© 2024 Fang H et al. *JAMA Network Open.*

eFigure 1. County-Year Records Cleaning Flow Diagram

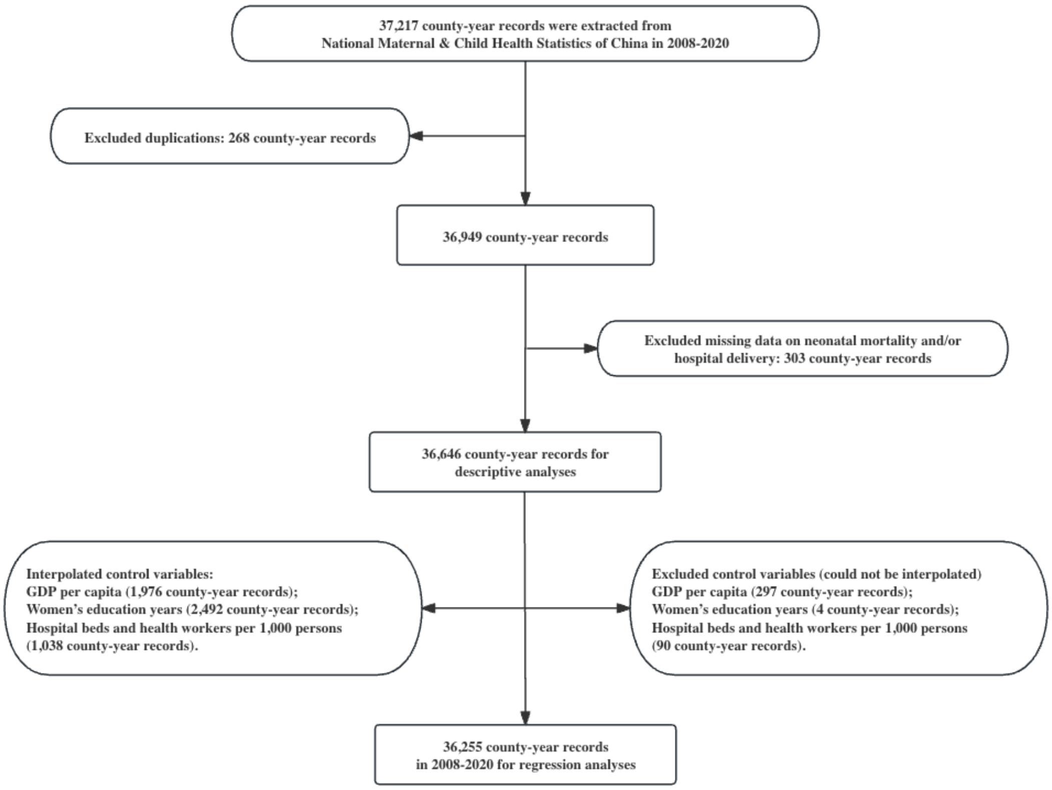

© 2024 Fang H et al. *JAMA Network Open.*

eFigure 2. Trends of Neonatal Mortality and Hospital Delivery From 2008 to 2020 by Four Quartiles of GDP per Capita

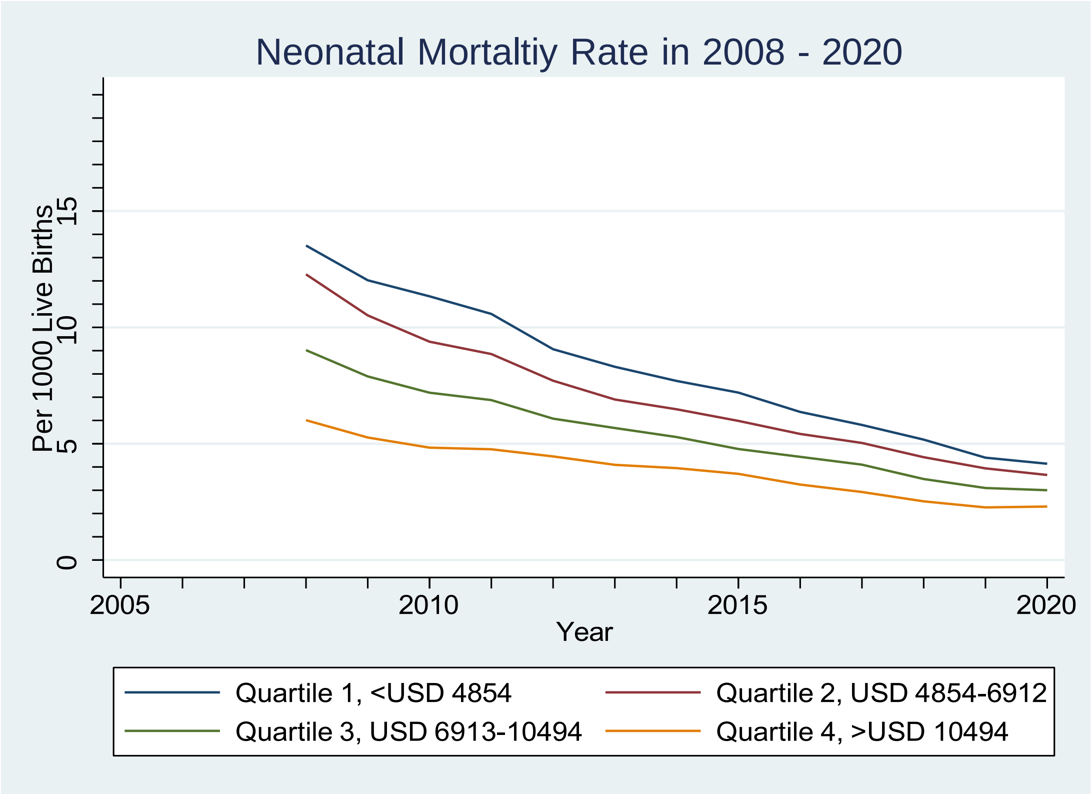

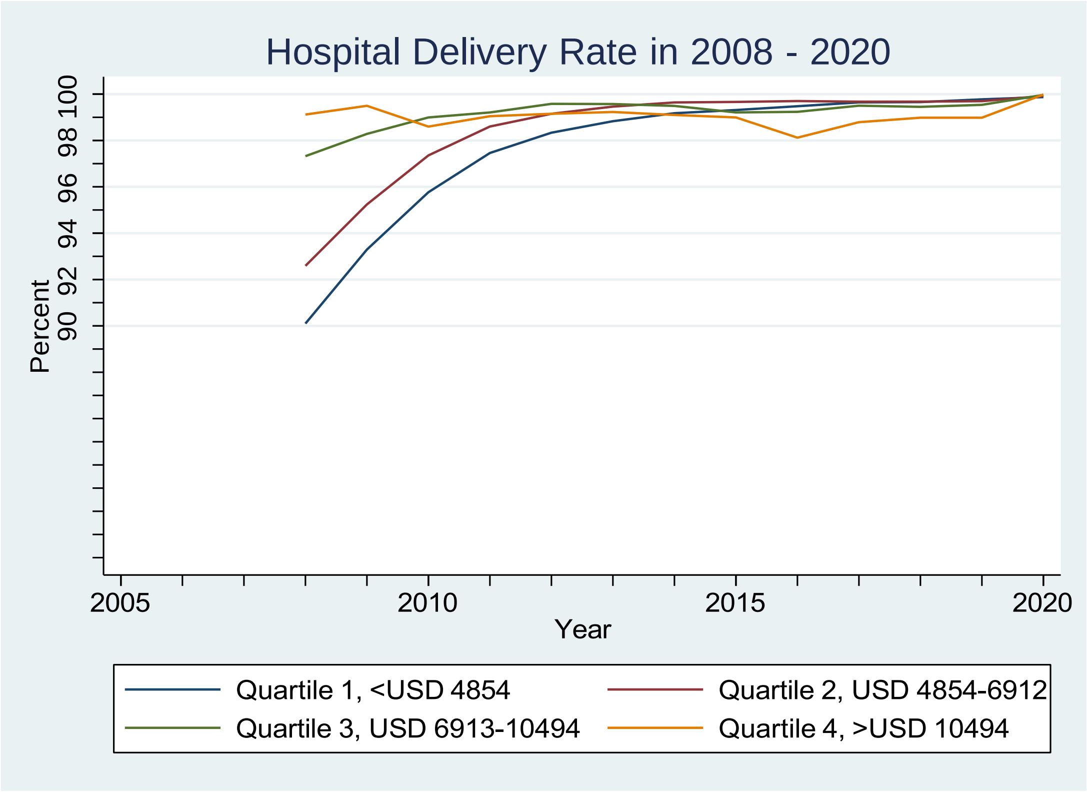

© 2024 Fang H et al. *JAMA Network Open.*
