# Supplementary material for: Regional Disparities, Economic Development, and Neonatal Mortality and Hospital Delivery in China
Source: JAMA Netw Open. 2024 Nov 6;7(11):e2443423. doi: 10.1001/jamanetworkopen.2024.43423 (PMC11541646; doi:10.1001/jamanetworkopen.2024.43423)
Supplement: Supplement 2. — Data Sharing Statement [file jamanetwopen-e2443423-s002.pdf]

Data Sharing Statement

Fang. Regional Disparities, Economic Development, and Neonatal Mortality and Hospital Delivery in China. *JAMA Netw Open*. Published November 06, 2024. doi:10.1001/jamanetworkopen.2024.43423

Data
Data available: No

Additional Information
Explanation for why data not available: Data presented in this study were obtained from the Office for National Maternal & Child Statistics affiliated with Peking University and National Health Commission of the People's Republic of China. We are not allowed to share these data due to existing data protection rules. The corresponding authors affirm that the manuscript is an honest, accurate, and transparent account of the study being reported; that no important aspects of the study have been omitted; and that any discrepancies from the study as planned (and, if relevant, registered) have been explained.
